# *Borrelia burgdorferi* induces a type I interferon response during early stages of disseminated infection in mice

**DOI:** 10.1186/s12866-016-0644-4

**Published:** 2016-03-08

**Authors:** Mary M. Petzke, Radha Iyer, Andrea C. Love, Zoe Spieler, Andrew Brooks, Ira Schwartz

**Affiliations:** Department of Microbiology and Immunology, New York Medical College, Valhalla New York, USA; Bionomics Research and Technology Center, Robert Wood Johnson-University of Medicine and Dentistry of New Jersey/Rutgers University, Piscataway, New Jersey USA; Present Address: Nexcelom Bioscience LLC, Lawrence, Massachusetts USA

**Keywords:** Lyme disease, Type I interferon, Bacterial pathogenesis, Mouse model

## Abstract

**Background:**

Lyme borrelia genotypes differ in their capacity to cause disseminated disease. Gene array analysis was employed to profile the host transcriptome induced by *Borrelia burgdorferi* strains with different capacities for causing disseminated disease in the blood of C3H/HeJ mice during early infection.

**Results:**

*B. burgdorferi* B515, a clinical isolate that causes disseminated infection in mice, differentially regulated 236 transcripts (P < 0.05 by ANOVA, with fold change of at least 2). The 216 significantly induced transcripts included interferon (IFN)-responsive genes and genes involved in immunity and inflammation. In contrast, *B. burgdorferi* B331, a clinical isolate that causes transient skin infection but does not disseminate in C3H/HeJ mice, stimulated changes in only a few genes (1 induced, 4 repressed). Transcriptional regulation of type I IFN and IFN-related genes was measured by quantitative RT-PCR in mouse skin biopsies collected from the site of infection 24 h after inoculation with *B. burgdorferi*. The mean values for transcripts of *Ifnb*, *Cxcl10*, *Gbp1*, *Ifit1*, *Ifit3*, *Irf7*, *Mx1*, and *Stat2* were found to be significantly increased in *B. burgdorferi* strain B515-infected mice relative to the control group. In contrast, transcription of these genes was not significantly changed in response to *B. burgdorferi* strain B331 or B31-4, a mutant that is unable to disseminate.

**Conclusions:**

These results establish a positive association between the disseminating capacity of *B. burgdorferi* and early type I IFN induction in a murine model of Lyme disease.

## Background

Lyme disease, the most common vector-borne infection in North America [1], is caused by the spirochete *Borrelia burgdorferi* and transmitted through the feeding of infected *Ixodes* species ticks [2]. Approximately 70–80 % of patients develop a characteristic skin lesion, erythema migrans (EM), at the site of inoculation that is characterized by an influx of immune cells, predominantly T lymphocytes, macrophages/monocytes, and dendritic cells [3, 4]. If left untreated with recommended antibiotic therapy [5], sequelae of Lyme disease can include rheumatologic, neurologic and cardiac symptoms following hematogenous dissemination of the spirochete from the site of inoculation in the skin to target tissues such as the joints, central nervous system and heart [6].

The potential for dissemination is likely dependent on multiple host and pathogen factors, including the existence of diverse *B. burgdorferi* genotypes that can be classified based on a number of molecular characteristics, including restriction fragment-length polymorphism of the 16S-23S ribosomal DNA spacer region (ribosomal spacer type; RST) and the sequence of outer surface protein C (OspC) [7]. Numerous studies have established an association between genotype and the capacity to cause invasive disease. RST1 strains are more likely to cause disseminated infection in Lyme disease patients, whereas RST2 and RST3 strains are less frequently detected in the blood [8–11]. Four of the 16 identified OspC genotypes were found to account for 80 % of cases of disseminated Lyme disease in the Northeastern United States, including both of the OspC genotypes corresponding to RST1 (OspC A and B); in contrast, only one of the four OspC genotypes associated with RST2 (OspC K), and one of the ten OspC genotypes associated with RST3 (OspC I), were identified as highly invasive [11]. These correlative data were confirmed by direct experimental validation using a murine model of Lyme borreliosis; infection of C3H/HeJ mice with RST1 strains resulted in significantly higher spirochete loads in tissue, and more severe arthritis and carditis, than did infection with RST3 isolates, some of which did not disseminate from the inoculation site [12, 13].

*B. burgdorferi* elicits the production of both pro- and anti-inflammatory cytokines via recognition of spirochetal cellular components by cells of the host’s innate immune system [14–17]. The induced cytokine profile may have a critical impact on disease outcome, as a strong pro-inflammatory response early in infection appears to mediate host protection in both mice and in Lyme disease patients [4, 18–21]. Intriguingly, expression profiling of a murine macrophage cell line stimulated with diverse *B. burgdorferi* clinical isolates revealed no genotype-specific differences in mRNA or protein levels for a number of pro-inflammatory cytokines known to be associated with Lyme disease pathogenesis [22]. Similar results were observed when comparing *B. burgdorferi* clinical isolates of varying genotype using an *ex vivo* human peripheral blood mononuclear cell (PBMC) model and measuring secreted cytokine proteins [23]. However, the latter study identified a correlation between the induction of IFN-α, a type I interferon (IFN), and *B. burgdorferi* genotype; significantly higher levels of IFN-α were elicited by strains with a greater capacity for dissemination [23]. This finding was corroborated by a separate study in which RST1 isolates induced significantly greater IFN-α production by human PBMCs relative to RST3 isolates [24]. Type I IFNs have also been detected in serum and in blister fluids raised over EM lesions of Lyme disease patients and serum levels of IFN-α were found to be significantly higher in patients with multiple EM skin lesions, an indication of disseminated Lyme disease, relative to patients with a single EM lesion [4]. In addition, type I IFNs and IFN-responsive genes are expressed in the joints of *B. burgdorferi*-infected mice and play a crucial role in the subsequent development of Lyme arthritis [25, 26].

The present study was designed to identify host transcriptional alterations, including those of IFN-responsive genes, elicited in a murine model of Lyme borreliosis. Gene array analysis was employed to profile global gene expression changes induced by *B. burgdorferi* strains of varying genotypes in mouse whole blood at 14 days post-inoculation. Additionally, transcription of type I IFNs and IFN-related genes was measured 24 h post-infection in mouse skin at the site of intradermal inoculation with various *B. burgdorferi* strains.

## Results

### Differential infection outcomes in mice inoculated with *B. burgdorferi* clinical isolates

**Fig. 1:**
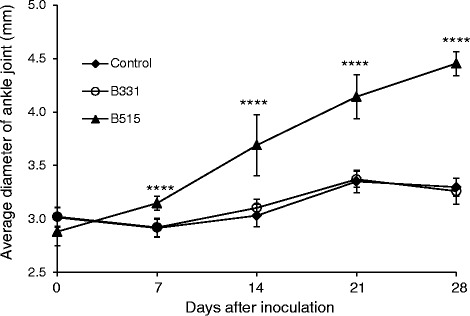
Arthritis development in infected mice. Groups of 15 C3H/HeJ mice were inoculated intradermally by needle with 1×10^4^ spirochetes of *B. burgdorferi* strains B515 or B331, or with medium (control). Tibiotarsal joints were measured on days 0, 7, 14, 21, and 28 post-infection. Measurements are shown as the mean value of each experimental group, with bars indicating the standard deviation. ****, *P* < 0.0001 relative to the control and B331-infected groups

C3H/HeJ mice were inoculated with *B. burgdorferi* B515, an RST1 strain that causes disseminated infection in mice [13] or *B. burgdorferi* B331, an RST3A strain that is unable to disseminate from the skin [13]. Forty-four (97.8 %) of the 45 blood and tissue samples collected on day 28 from mice infected with *B. burgdorferi* B515 were positive by culture (Table 1). In contrast, spirochetes could not be recovered from any tissues collected from mice infected with *B. burgdorferi* B331 (0/60; 0 %). Notably, culture positivity from all individual tissues assessed was significantly higher for mice infected with *B. burgdorferi* B515 as compared with *B. burgdorferi* B331 (*P* < 0.001) (Table 1). To confirm disseminated infection in B515-infected mice, development of joint swelling as an indicator of clinically apparent arthritis was monitored by measurement of the ankle-joint diameter using a digital caliper on days 7, 14, 21 and 28 post-infection. The average ankle joint diameters were significantly greater for mice infected with *B. burgdorferi* B515 as compared to mice infected with *B. burgdorferi* B331 or medium-inoculated controls at each of these time points (*P* < 0.0001) (Fig. 1). Taken together, these results confirm the development of disseminated infection in mice inoculated with *B. burgdorferi* B515, but not in *B. burgdorferi* B331-inoculated animals.

**Table 1:** Culture of *B. burgdorferi* from tissue specimens of infected mice

	Number of samples with positive cultures/number tested (%)				
	Day 14	Day 28			
	Ear	Ear	Skin ^*a*^	Bladder	Joint^*b*^
B515^*c*^	15/15 (100)	12/12 (100)^*d*^	13/14 (92.9)	14/14 (100)	5/5 (100)
B331	0/15 (0.0)	0/15 (0)	0/15 (0.0)	0/15 (0)	0/5 (0)

^*a*^Skin biopsy was collected from the inoculation site

^*b*^Joints were cultivated from one mouse per subgroup

^*c*^One mouse in the B515-infected group died between Day 14 and Day 28

^*d*^Two samples were contaminated

### *B. burgdorferi* elicits genotype-dependent transcriptional profiles in mouse blood

**Fig. 2:**
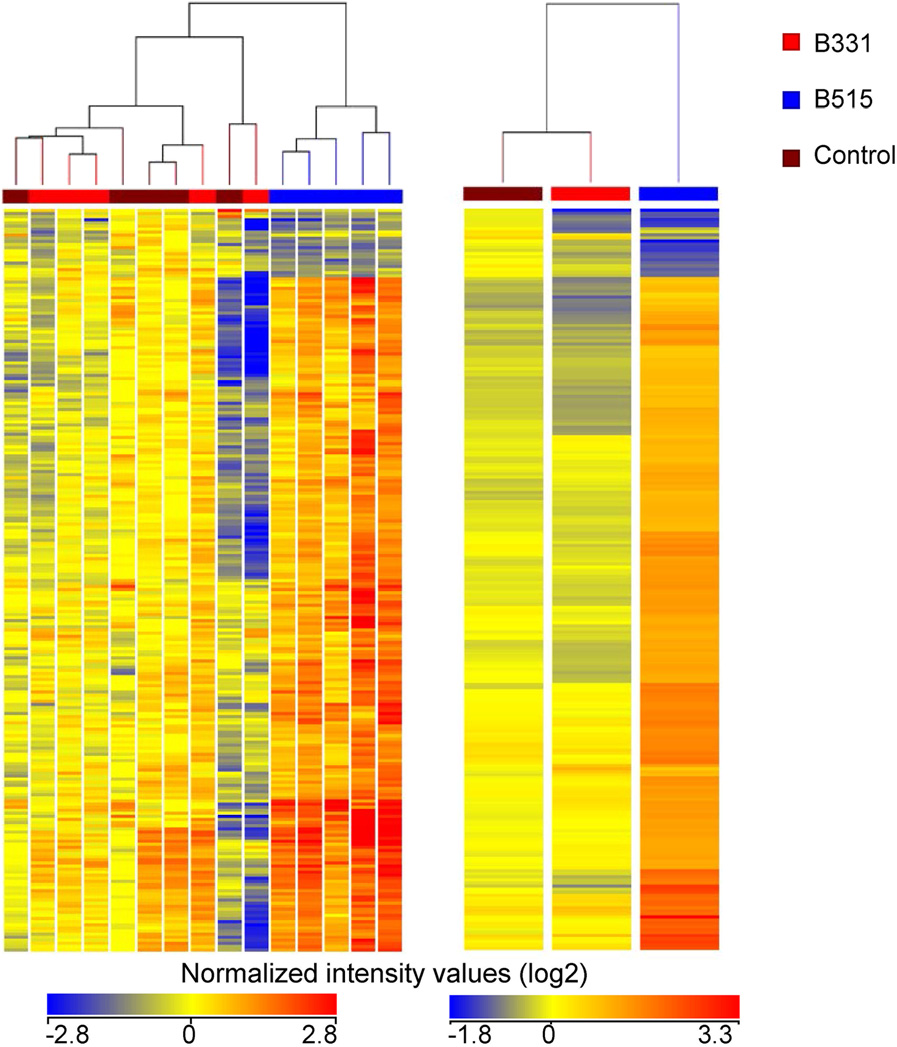
Hierarchical clustering of differentially regulated probe sets in *Borrelia*-infected mouse blood. A total of 239 probe transcripts were defined as differentially regulated (*P* < 0.05 and fold change of at least 2 relative to the medium-inoculated controls) in mouse blood at day 14 post-inoculation with *B. burgdorferi* strains B515 or B331. Differentially regulated probe sets were subjected to unsupervised hierarchical clustering based on transcript and experimental condition. Results are displayed as heat maps of the log of the normalized intensity values for individual samples (left) or averaged values for each experimental group (right)

Gene array analysis was performed in order to examine the host global transcriptome changes elicited by *B. burgdorferi* in blood. RNA was isolated from whole blood collected on day 14, when disseminated infection could be confirmed by culture of ear biopsies. A total of 239 transcripts was defined as differentially regulated based on having a *P* value of <0.05 and fold-change of at least 2 relative to the control group. The response to *B. burgdorferi* B515 included 236 differentially regulated transcripts (216 induced, 20 repressed). In contrast, *B. burgdorferi* B331 stimulated changes in relatively few transcripts (1 induced, 4 repressed); this result was not unexpected, as spirochetes could not be recovered from the tissues of any of the *B. burgdorferi* B331-infected mice (Table 1). The 239 differentially regulated transcripts were subjected to hierarchical clustering based on both transcript (probe set) and experimental condition (Fig. 2). Samples from mice that had been infected with *B. burgdorferi* B515 displayed similar transcriptional profiles and clustered together. In contrast, samples from *B. burgdorferi* B331-infected mice formed a second cluster together with samples from medium-inoculated control animals.

To validate the expression values generated by gene array, transcript levels for five genes were assessed by real-time RT-PCR (Table 2). Although the absolute values obtained by gene array and RT-PCR varied, a similar trend in transcriptional expression was observed by both methods for all of the genes assayed. In summary, these findings demonstrate that transcriptional changes in mouse blood vary according to the infecting *Borrelia* strain.

**Table 2:** Real-time RT-PCR validation of gene array results

			B515		B331	
Probe set ID	Gene symbol	Gene name	Array^a^	RT-PCR^a^	Array	RT-PCR
1419691_at	Camp	cathelicidin antimicrobial peptide	4.0	6.5	−1.1	−1.2
1418722_at	Ngp	neutrophilic granule protein	5.6	25.6	−1.2	1.8
1420549_at	Gbp1	guanylate binding protein 1	1.9	−1.1	1.1	−1.7
1427256_at	Vcan (Cspg2)	versican	4.1	12.2	1.2	1.9
1423466_at	Ccr7	chemokine (C-C motif) receptor 7	−1.9	−1.2	−1.2	1.0

^a^Mean fold change values relative to medium-inoculated control group

### *B. burgdorferi* induces expression of inflammatory and anti-microbial transcripts during disseminated infection including IFN-responsive genes

Selected induced and repressed transcripts involved in the immune response are presented (Tables 3 and 4). *B. burgdorferi* B515 elicited elevated expression of genes with defined roles in antimicrobial defense, remodeling of the extracellular matrix, dermal inflammatory conditions, innate immunity, B cell activation and the development of humoral immunity (*Ighg*) and the generation of tolerogenic dendritic cells (*Clec4a2*) [27]. Among the most highly induced transcripts were lactotransferrin (*Ltf*); cathelicidin antimicrobial peptide (*Camp*) and its receptor, formyl peptide receptor 2 (*Fpr2*); versican (*Vcan*); chitinase 3-like 3 (*Chi3l3)*; and lipocalin 2 (*Lcn2*). Also included was the gene encoding TLR13, which has recently been identified as a receptor for bacterial ribosomal RNA [28–30], as well as genes encoding two proteins (TLR2 and CD14) with established roles in the innate immune response to *B. burgdorferi* lipoproteins [31–33]. Two of the induced transcripts (*Ifitm2* and *Ifitm6*) contained the term “interferon induced” in the gene title. A database search for interferon-responsive genes identified 92 of the 216 (42.6 %) B515-induced transcripts as interferon responsive (http:www.interferome.org).

**Table 3:** Transcripts significantly induced in mouse blood in response to *B. burgdorferi*

Unigene	Gene symbol	Gene title	Gene ontology	FC^a^B515	FC^a^B331
Transcripts induced by *B. burgdorferi* B515					
Mm. 387173	Chi3l3	chitinase 3-like 3	Inflammatory response	9.14	1.17
Mm.9537	Lcn2	lipocalin 2	Response to virus	7.25	−1.43
Mm.236225	Ngp	neutrophil granule protein	Defense response to bacterium	5.64	−1.20
Mm.16415	Mmp8	matrix metallopeptidase 8	Proteolysis	5.21	1.01
Mm.1349	Il1r2	Interleukin 1 receptor, type II	Lipid metabolic process	4.78	−1.02
	Ighg	Immunoglobulin heavy chain (gamma polypeptide)		4.23	−1.08
Mm.158700	Vcan	versican	Cell adhesion, heart development	4.10	1.20
Mm.248327	Clec4e	C-type lectin domain family 4, member e	Immune response	4.03	1.11
Mm.3834	Camp	cathelicidin antimicrobial protein	Defense response to bacterium	3.96	−1.09
Mm.274927	Ccr1	chemokine (C-C) receptor 1	Inflammatory response	3.68	−0.05
Mm.24208	Il13ra1	interleukin 13 receptor, alpha 1	Cytokine-mediated signaling pathway	3.63	1.15
Mm.477887	Fpr2	formyl peptide receptor 2	G-protein coupled receptor signaling	3.62	−1.35
Mm.282359	Ltf	lactotransferrin	Iron ion transport, homeostasis	3.60	−1.15
Mm.248360	Anxa1	annexin A1	Leukocyte homeostasis	3.45	−1.26
Mm.9277	Pla2g7	phospholipase A2, group VII	Inflammatory response	3.41	1.14
Mm.882	Il1rn	interleukin 1 receptor antagonist	Negative regulation of cytokine-mediated signaling	3.30	1.25
Mm.87596	Tlr2	toll-like receptor 2	Pattern recognition receptor signaling pathway	3.01	−1.12
Mm.276440	Ifitm6	interferon induced transmembrane protein 6	Response to biotic stimulus	3.00	−1.30
Mm.143718	Mefv	Mediterranean fever	Inflammatory response	2.93	1.09
Mm.19131	C3	complement component 3	Inflammatory response, innate	2.91	−1.04
Mm.486	Lamp2	lysosomal-associated membrane protein 2		2.89	−1.09
Mm.336203	Tlr13	toll-like receptor 13	Inflammatory response, signal Transduction	2.72	−1.22
Mm.3460	Cd14	CD14 antigen	Response to molecule of bacterial origin	2.60	−1.44
Mm.170515	Nfkbia	nuclear factor of kappa light polypeptide gene enhancer in B cells inhibitor, alpha	Protein import into nucleus, Translocation	2.55	−1.29
Mm.47384	Clec4a2	C-type lectin domain family 4, member a2	Immune response	2.48	1.10
Mm.379266	Ifitm2	interferon induced transmembrane protein 2	Response to biotic stimulus	2.41	−1.22
Transcript induced by *B. burgdorferi* B331					
Mm247642	Arap3	ArfGAP with RhoGAP domain, ankyrin repeat and PH domain	Signal transduction	1.71	2.02

^a^FC = fold change

Twenty transcripts were repressed by *B. burgdorferi* B515 (Table 4). Of these, two transcripts (*Pfn2*, profilin and *Tnfrsf13c*, tumor necrosis factor receptor superfamily, member 13c) were also repressed by *B. burgdorferi* B331. *Pfn2* has an annotated function in cytoskeleton organization and Tnfrsf13c is involved in B-cell homeostasis. Other transcripts involved in humoral immunity were repressed by B515; these included *Fcer2a* (Fc receptor, IgE, low affinity II, alpha polypeptide), *Ighv14-2* (immunoglobulin heavy variable 14–2), and *Ebf1* (early B cell factor).

**Table 4:** Transcripts significantly repressed in mouse blood in response to *B. burgdorferi*

Unigene	Gene symbol	Gene title	Gene ontology	FC^a^B515	FC^a^B331
Transcripts uniquely repressed by *B. burgdorferi* B515					
Mm.1233	Fcer2a	Fc receptor, IgE, low affinity II, alpha polypeptide	Positive regulation of humoral Immune response mediated by circulating immunoglobulin	−3.34	−1.42
	Ighv14-2	immunoglobulin heavy variable 14-2		−2.59	−1.65
Mm.431426	Bach2	BTB and CNC homology 2	Regulation of transcription, DNA-dependent	−2.42	−1.80
Mm.476307	Zfp799	zinc finger protein	Regulation of transcription, DNA-dependent	−2.40	−1.39
Mm.126193	Tnik	TRAF2 and NCK interacting kinase	Protein phosphorylation	−2.39	−1.40
Mm.439662	Ebf1	early B cell factor	Transcription, DNA-dependent	−2.30	−1.28
Mm.486382	Gm8369	predicted gene 8369		−2.34	−1.90
Mm.482376	4833423F13	RIKEN cDNA 4833423F13 gene		−2.33	−1.31
	Rik				
Mm.441077	BC002059	cDNA sequence BC002059		−2.33	−1.65
Mm.1850	Fcrla	Fc receptor-like A	Cell differentiation	−2.21	−1.15
Mm.3411	Orc2	origin recognition complex, subunit 2	DNA replication	−2.06	−1.73
Transcripts repressed by *B. burgdorferi* B515 and B331					
Mm.271744	Pfn2	profilin 2	Cytoskeleton organization	−3.14	−2.94
Mm.240047	Tnfrsf13c	tumor necrosis factor receptor	B-cell homeostasis	−2.10	−2.16
		superfamily, member 13c			
Transcripts uniquely repressed by *B. burgdorferi* B331					
Mm.41979	Mcpt8	mast cell protease 8	Proteolysis	−1.52	−2.60
Mm.273270	Cbll1	Casitas B-lineage lymphoma-like 1	Negative regulation of cell adhesion	−1.49	−2.30

^a^FC = fold change

### Induction of type I IFN and IFN-responsive genes by *B. burgdorferi* in skin correlates with capacity for dissemination

Expression of IFN-responsive genes has been shown to be a critical factor in the development of arthritis in a susceptible mouse model of Lyme borreliosis [25, 26]. The observation that a type I IFN response can be detected in the blood at day 14 post-inoculation, however, suggested a potential role for type I IFNs during earlier stages of infection. In order to evaluate the differential expression of type I IFN-related genes at the site of inoculation in mouse skin, groups of nine mice were inoculated with 1×10^4^ spirochetes of *B. burgdorferi* strains B515, B331, or B31–4, a derivative of type strain B31 (RST1) that lacks all linear plasmids, is unable to disseminate in mice [34] and does not induce a type I IFN response by human peripheral blood mononuclear cells [23]. Skin biopsies were collected 24 h post-inoculation for RNA isolation and tissues samples were collected at day 14 or day 28 for assessment of culture positivity. Spirochetes could be recovered from 24-h skin biopsies collected from the site of inoculation from all four (100 %) of the *B. burgdorferi* B515-infected mice and from at least one additional tissue from each of these mice. Interestingly, although 24-h skin biopsies from 75 % (3/4) of B331-infected mice were culture positive, spirochetes could not be recovered from any of the tissues from these mice. As expected, neither the 24-h skin biopsies nor the 14 or 28 day tissues collected from B31-4-inoculated mice were culture positive (Table 5). This indicates that inoculation with strain B331 resulted in transient infection at the injection site, but these spirochetes could not establish infection at distal tissue sites.

**Table 5:** Culture of *B. burgdorferi* from tissues of mice infected with strains B515, B331 or B31-4

Number of samples with positive cultures/number tested (%)						
	Day 1	Day 14	Day 28			
	Skin^a^	Ear	Ear	Skin^a^	Bladder	Joint
B31-4	0/4 (0)	0/9 (0)	0/9 (0)	0/9 (0)	0/9 (0)	0/9 (0)
B331	3/4 (75.0)	0/9 (0)	0/9 (0)	0/9 (0)	0/9 (0)	0/9 (0)
B515^b^	4/4 (100.0)	5/6 (83.3)	5/6 (83.3)	3/6 (50.0)	5/6 (83.3)	5/6 (83.3)

^a^Skin biopsy was collected from the inoculation site

^b^Three mice in the B515-infected group died of causes unrelated to the infection

**Fig. 3:**
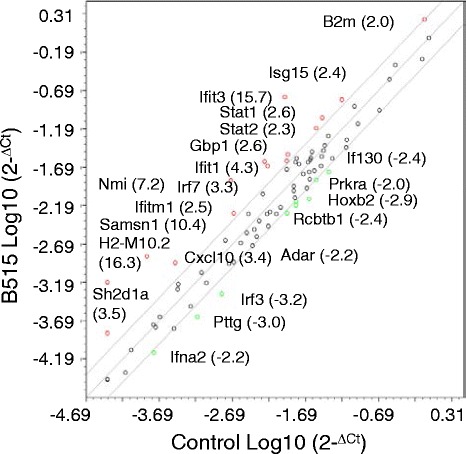
Expression profiling of type I IFN-related genes in B515-infected mouse skin. Skin biopsies were collected from the site of inoculation 24 h after infection of mice with 1×10^4^ *B. burgdorferi* B515 spirochetes or with medium only (Control). Equivalent amounts of RNA from five animals per experimental group were separately pooled and used as a template for RT-PCR using the RT^2^ Profiler PCR Array PAMM-016: Mouse Interferon α, β Response kit. Gene symbols are used to designate transcripts that were found to be changed at least two-fold in B515-infected skin relative to skin from controls. Fold change values are indicated in parentheses

RNA was isolated from skin biopsies of five mice that were either mock-infected or inoculated with *B. burgdorferi* strain B515. These RNAs were separately pooled and used as templates for RT-PCR using the RT^2^ Profiler PCR Array PAMM-016: Mouse Interferon α,β Response kit (SABiosciences). This kit profiles the expression of 84 genes involved in the IFN-α and IFN-β immune response, including signaling molecules, IFN-responsive genes, and genes associated with interferon resistance. Fifteen genes were induced >2-fold in *B. burgdorferi* B515-infected mouse skin relative to the medium-inoculated control group (Fig. 3). These genes included *Ifit3* (15.7-fold), *Ifit1* (4.3-fold), *Cxcl10* (3.4-fold), *Irf7* (3.3-fold), *Gbp1* (2.6-fold), and *Stat2* (2.3-fold).

**Fig. 4:**
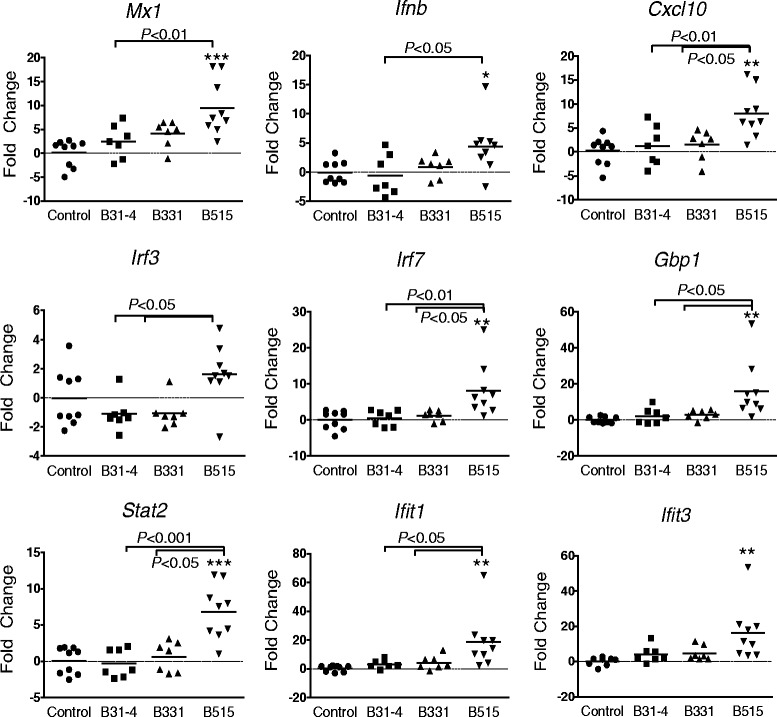
IFN-related genes are induced at the site of inoculation in response to a disseminating *B. burgdorferi* strain. Groups of C3H/HeJ mice were inoculated with 1x10^4^ spirochetes of *B. burgdorferi* strains B515 (*n* = 9), B331 (*n* = 7) or B31-4 (*n* = 7), or with medium (control; *n* = 9). After 24 h skin biopsies were collected from the site of inoculation and processed for RNA. Transcript levels for nine IFN-responsive genes were assessed for individual animals by real-time RT-PCR. The mean fold change value for each experimental group was calculated relative to the mean value of the control group and is indicated by a horizontal line. *, *P* < 0.05; **, *P* < 0.01; ***, *P* < 0.001 relative to the mean of the control group

Based on these PCR array results, differential induction of type I IFNs and selected IFN-responsive genes by *B. burgdorferi* was further evaluated by real-time RT-PCR analysis of RNA isolated from 24-h skin biopsies collected from individual animals. RNA was obtained from all nine of the mice in each of the *B. burgdorferi* B515-infected and control groups, and from seven animals each in the groups that had been infected with *B. burgdorferi* strains B31-4 and B331. Transcript levels were assessed for genes encoding murine IFN-β (*Ifnb*) and the IFN-responsive genes *Cxcl10*, *Mx1*, *Irf3*, *Irf7*, *Gbp1*, *Stat2*, *Ifit1*, and *Ifit3*. With the exception of *Irf3*, the mean fold-change values for all genes assessed were found to be significantly elevated in the skin of *B. burgdorferi* B515-infected mice relative to the medium-inoculated control group: *Mx1* (9.5-fold), *Ifnb* (4.4-fold), *Cxcl10* (8.0-fold), *Irf7* (8.1-fold), *Gbp1* (16.0-fold), *Stat2* (6.8-fold), *Ifit1* (18.8-fold), and *Ifit3* (16.2-fold) (Fig. 4). In contrast, none of the genes assessed was found to be significantly induced or repressed relative to the controls in the mice inoculated with either *B. burgdorferi* strains B31-4 or B331. All genes except *Ifit3* were significantly induced in *B. burgdorferi* B515-infected mice relative to *B. burgdorferi* B31-4-inoculated mice (*P* < 0.01). In addition, transcript levels for *Cxcl10*, *Irf3*, *Irf7*, *Gbp1*, *Stat2*, and *Ifit1* were also significantly higher in skin at the inoculation site of mice infected with *B. burgdorferi* strain B515 as compared to animals infected with *B. burgdorferi* strain B331 (*P* < 0.05). These results demonstrate that only the *B. burgdorferi* strain capable of causing disseminated infection induces significant expression of type I IFN-related genes in mouse skin during early infection, in contrast to strains that do not disseminate.

## Discussion

In this study we utilized host transcriptional profiling to identify alterations in gene expression that occur following intradermal inoculation of *B. burgdorferi* in a murine infection model. For a strain of *B. burgdorferi* that causes disseminated infection, a number of IFN-inducible genes were expressed both during acute localized infection at the inoculation site in skin and in blood collected 14 days after infection. This finding is consistent with numerous studies demonstrating an association between the induction of IFNs and Lyme disease pathogenesis. In human patients, significantly higher expression of serum IFN-α protein is associated with disseminated infection, as defined by the presence of multiple EM skin lesions as compared to patients with a single EM [4]. Transcripts for IFN-β are induced in human monocytes and PBMCs during co-culture with *B. burgdorferi* [35, 36]. Recently, we demonstrated that *B. burgdorferi* clinical isolates which are more frequently associated with disseminated infection in mice and humans induce significantly higher levels of IFN-α protein in human PBMCs as compared with isolates that are less likely to disseminate [23]. The current results extend the latter findings by identifying a correlation between *B. burgdorferi* dissemination and the induction of a type I IFN response in mice.

Factors other than spirochete density appear to be critical for the induction of an IFN-responsive gene expression profile. Although viable spirochetes of *B. burgdorferi* strain B331 could be cultured from 24-h skin biopsies, expression for many of the IFN-responsive genes was significantly lower than that induced by an equivalent inoculum of *B. burgdorferi* B515 (Fig. 4). This result indicates that the magnitude of IFN-responsive gene expression is not only dependent on the presence of live spirochetes, but may also be an intrinsic genomic property of the *B. burgdorferi* isolate. Since quantitative assessment of spirochete burden by qPCR was not performed, no comparison can be made of the relative numbers of spirochetes present in the skin 24 h after infection. While this is a limitation of the current study, the major goal was to assess a correlation between expression of type I IFN-related genes and the potential for dissemination, which requires viable spirochetes; qPCR detects DNA from both live and dead spirochetes [37]. Moreover, several recent studies have found that spirochete DNA present in infected mouse skin is close to the limit of detection by qPCR prior to 72 or 96 h following inoculation, even when using DNA extracted from a much larger quantity of skin than was collected in this study [38, 39].

The results demonstrate that a *B. burgdorferi* strain that causes disseminated infection induces the expression of IFN-responsive genes in the skin and blood of infected animals. Previous studies had shown that IFN-responsive gene expression is a crucial factor in the development of Lyme arthritis in C3H mice [26]. The current findings indicate that these gene products might have additional roles during earlier stages of infection. Interestingly, despite the induction of IFN-responsive gene transcripts by *B. burgdorferi* B515 in blood, transcripts for the interferons themselves were not detected by gene array analysis. This could be due to the fact that dendritic cells, the classic type I IFN-producing cell type, account for a very small percentage of the cellular components of blood relative to the number of cells that are capable of expressing IFN-responsive genes. Alternatively, as the presence of IFN transcripts in the blood was not assessed by RT-PCR, the possibility that IFN transcript levels were below the threshold of detection by gene array cannot be excluded. It should be noted that a gene array analysis of human dermal fibroblasts exposed to three different strains of *B. burgdorferi* showed induction of the IFN-responsive gene transcripts *CXCL10*, *IRF1*, *STAT1*, *STAT2*, *OAS2*, and *IFIH1*, but no induction of any IFN [40]. In addition, IFNs were not detectable by gene array analysis in the ankle tissue of *B. burgdorferi*-infected mice which developed severe arthritis concomitant with the expression of numerous IFN-responsive genes [25].

In addition to IFN-related transcripts, the transcripts that were most highly induced by *B. burgdorferi* B515 in mouse blood included a cluster of genes with defined roles in antimicrobial defense, remodeling of the extracellular matrix, dermal inflammatory conditions, and dendritic cell functions, particularly in skin (Table 3). Camp, an antimicrobial peptide with multiple functions in innate immunity of the skin and other epithelial barriers, is exploited by *Staphylococcus aureus* to promote bacterial dissemination and the establishment of invasive infection [41]. Formylpeptide receptor 2 (Fpr2), the receptor for Camp [42], was also induced by *B. burgdorferi* B515 (Table 3). Murine chitinase 3-like protein 3 (Chi3L3) is a substrate for several matrix metalloproteases, including MMP-9, and is upregulated in the EM skin lesions of patients with acute Lyme disease [43]. Versican (Vcan) mediates extracellular matrix remodeling by promoting the interaction of pro-MMP-9 with gelatin and collagen [44].

The observation that *B. burgdorferi* B515 induced the expression of IFN-related genes in blood at 14 days post-inoculation suggested that such a response might occur at the initial infection site. Indeed, skin biopsies collected 24 h after inoculation of mice with *B. burgdorferi* strain B515, but not with *B. burgdorferi* strains B331 or B31-4, showed induction of a number of IFN-responsive genes. These same genes were among those induced in human PBMCs that had been exposed *ex vivo* to *B. burgdorferi* B515 [17, 23]. The presence of an IFN-responsive gene profile in mouse skin biopsies leads to the question of the identity of the inducing cytokine, as many IFN-responsive genes can be induced by more than one class of IFN. In the current study, transcript levels for IFN-β were significantly induced, but transcription of IFN-γ was not assessed. *B. burgdorferi* is a potent inducer of IFN-γ, and both type I IFN and IFN-γ are produced in the EM lesion blister fluids of human Lyme disease patients [4]. In *B. burgdorferi*-stimulated human PBMCs, transcript levels for IFN-γ were observed to be at least 10-fold higher than levels for either IFN-α or IFN-β [36]. While we cannot exclude the possibility that the IFN-responsive gene expression profile observed in 24-h skin biopsies from mice infected with *B. burgdorferi* strain B515 is elicited by contributions from both IFN-γ and IFN-β, it should be noted that all strains, regardless of genotype, were equally effective at inducing high levels of IFN-γ protein, while only strains of the RST1 genotype elicited significantly higher production of IFN-α [23].

A limitation of the present study is that responses to only three *B. burgdorferi* isolates were studied. *B. burgdorferi* strain B515 is an RST1/OspC type A genotype; numerous studies have revealed that strains belonging to this genotype are genetically homogeneous, in contrast to RST2 and RST3 genotypes [45–48]. Moreover, *B. burgdorferi* RST1 isolates have been shown to induce significantly higher levels of IFN-α, and possess a greater capacity to cause invasive infection, than do RST3 isolates [23, 24]. It is therefore reasonable to predict that the findings of the present study with regard to induction of IFN-responsive genes in a genotype-specific manner would pertain to other *B. burgdorferi* isolates.

In the present study, mice were infected by needle inoculation. This procedure allows for the standardization of parameters, such as the number of spirochetes in the inoculum and the time of inoculation, and has been routinely employed to investigate the early kinetics of *B. burgdorferi* infection [38, 39]. A limitation of this approach is that it does not fully recapitulate physiological infection by tick bite. *Borrelia afzelii*-induced IFN signaling by mouse dendritic cells was suppressed by *Ixodes ricinus* tick saliva *in vitro*, although the amount of *Ifnb* transcript was not affected [49]. Tick salivary gland extracts were found to have a cytotoxic effect on human dermal fibroblasts *in vitro* [40], whereas tick salivary gland lysates enhanced spirochete load in tissues of infected mice [50]. In view of these findings, it would be of interest in future studies to address the effects of tick-related factors on the expression of IFN-responsive genes in the skin by comparing the results of needle inoculation with those obtained following spirochete inoculation via tick bite.

Induction of multiple IFN-responsive genes has been observed in a range of skin pathologies, including autoimmune skin inflammation [51], wound healing following skin injury [52], and the development of adult and juvenile dermatomyositis [53]. While predominantly host protective during viral infections, type I IFNs have also been shown to promote the pathogenesis of several intracellular and extracellular bacteria, including species that invade epithelial barriers [54–58]. One proposed mechanism for type I IFN-mediated bacterial pathogenesis occurs via suppression of myeloid cells [59]. Indeed, we have demonstrated that *B. burgdorferi* B31-A3, an RST1 strain that readily disseminates in mice, induces significantly higher expression of both IFN-α [23] and indoleamine 2,3-dioxygenase [60], an enzyme with immunomodulatory functions in human myeloid and plasmacytoid dendritic cells [61], as compared to a mutant strain that is severely attenuated for infectivity. Expression of indoleamine 2,3-dioxygenase was found to be driven by the concerted actions of both *B. burgdorferi*-induced IFN-γ and type I IFN [60]. The expression of type I IFN and IFN-responsive genes in mouse skin may therefore comprise a fingerprint of activated suppressor dendritic cells, which potentially could be manipulated by the spirochete in order to promote pathogenesis.

## Conclusions

Host transcriptional profiling was utilized to identify transcriptional alterations in blood and skin during *B. burgdorferi* infection in a murine infection model. The results suggest a number of potential host-protective and pathogen-promoting strategies that include the expression of antimicrobial peptides and molecules involved in extracellular matrix remodeling. Most notably, a strong association between early expression of *Ifnb* and IFN-responsive genes at the site of inoculation, and the capacity of certain *B. burgdorferi* strains to invade the bloodstream was observed. The findings imply that type I IFNs may play a pivotal role in the early stages of Lyme disease pathogenesis and suggests a potential immune evasion strategy that may be exploited by certain *B. burgdorferi* strains to facilitate dissemination.

## Methods

### *B. burgdorferi* strains and clinical isolates

*B. burgdorferi* isolates B515 (RST1) and B331 (RST3A) were obtained from skin biopsies taken from the EM skin lesions of Lyme disease patients enrolled in a prior study at the Lyme Disease Diagnostic Center at New York Medical College, Valhalla, NY and have been previously described [13]. *B. burgdorferi* strain B31-4, a high passage derivative of *B. burgdorferi* strain B31 (RST1) that lacks all linear plasmids and consequently is unable to disseminate in mice or induce IFN-α by human peripheral blood mononuclear cells, has been previously described [23, 34].

*B. burgdorferi* strains were grown at 37°C in modified Barbour-Stoenner-Kelly (BSK-S) medium [62]. Cultures were maintained at 37°C until spirochetes reached the mid- to late-log phase of growth (4×10^7^ to 8×10^7^/ml). In the experiment performed to assess induction of IFN transcripts in skin, *B. burgdorferi* cultures were initially grown as above. However, to simulate the temperature changes that occur during transmission from tick to mammal and that may enhance the expression of certain virulence factors [63, 64], spirochetes were subcultured and grown at 25 °C to the mid-log phase of growth, then diluted to 1 × 10^6^ spirochetes/ml and grown at 37 °C until cultures once again reached mid- to late-log phase of growth [65]. Spirochetes were enumerated and assessed for motility using dark-field microscopy [66].

### Mouse infection

All animal experimentation was conducted in strict accordance with the recommendations in the Guide for the Care and Use of Laboratory Animals of the National Institutes of Health. The protocols were approved by the Institutional Animal Care and Use Committee of New York Medical College.

C3H/HeJ mice were employed throughout, as this strain is susceptible to the development of Lyme arthritis and produces a type I IFN response to *B. burgdorferi* infection [12, 13, 26]. For assessment of the host transcriptional response in whole blood, specific pathogen-free 6-8-week-old male or female C3H/HeJ mice were randomly divided into groups of 15. Anesthetized mice were needle inoculated by intradermal injection on the shaven back with 1x10^4^ spirochetes of B515 or B331 in 0.1 ml BSK-S. Control animals were inoculated with 0.1 mL BSK-S alone. Whole blood samples were collected from each mouse at day 0 and day 14. Ear samples were obtained at day 14, and blood, ear, joint and urinary bladder tissues were collected following euthanization on day 28.

To assess infectivity, blood and tissue samples were cultured and serology was performed. Three to five drops of whole blood were added directly to 4 ml of BSK-S medium. Tissue samples (~10-20 mg) were placed in 70 % ethanol for 3 min and transferred to 4.5 mL of BSK-S containing fosfomycin (20 μg/mL), rifampicin (50 μg/mL), and amphotericin (2.5 μg/mL) (Sigma-Aldrich, all) to inhibit the growth of commensal bacteria [67]. Cultures were maintained at 37 °C for 4 weeks and examined weekly for the presence of spirochetes. Serum collected at days 14 and 28 was probed for the presence of anti-*Borrelia* antibodies using strain-specific whole-cell lysates [68]. Tibiotarsal joints were measured on days 7, 14, 21 and 28 using a digital metric caliper through the thickest anteroposterior diameter of the ankle.

In order to assess the type I IFN response in mouse skin during early infection, groups of nine C3H/HeJ mice were needle inoculated as described above with 1x10^4^ spirochetes of *B. burgdorferi* strains B515 or B331. As a control, a group of mice was infected with 1x10^4^ spirochetes of *B. burgdorferi* strain B31-4. This mutant derivative of *B. burgdorferi* strain B31 (RST1, OspC A) lacks all linear plasmids, is unable to disseminate in mice [34], and is unable to elicit IFN-α secretion by human immune cells [23]. Skin biopsies (~2 mm diameter) were collected from the injection site 24 h after inoculation. The skin biopsy was cut in half; one half was snap frozen and stored at −80 °C for RNA isolation and the other half was washed with 70 % ethanol, transferred to 5 mL of BSK-S containing the antibiotics described above and incubated. To assess spirochetal dissemination, ear, joint, and urinary bladder tissues, and skin collected from the site of inoculation, were collected following euthanization at day 28 and assessed for the growth of spirochetes as described above.

### RNA isolation from murine skin biopsies

For real time RT-PCR analysis of individual IFN-responsive genes, skin samples were homogenized in a glass tissue homogenizer containing 300 μL of denaturation solution from the Ambion ToTALLY RNA isolation kit (Life Technologies, Grand Island, NY). Mouse hair was removed from the samples by centrifugation at 3000 rpm for 3 min and RNA was isolated from skin homogenates using the Ambion ToTALLY RNA kit (Life Technologies). Contaminating genomic DNA was removed by on-column DNase I treatment using the Qiagen RNeasy Mini Kit (Qiagen, Valencia, CA). Total RNA was eluted in 30 μL of RNase/DNase-free water and quantitated by spectrophotometry (BioPhotometer, Eppendorf, Hauppauge, NY). RNA samples were stored at −80°C after the addition of 0.8 μL (32 U) of RNasin (Promega, Madison, WI).

### Microarray hybridization and data analysis

Whole blood was collected from mice at 14 days post-inoculation. In order to ensure a sufficient RNA yield, samples from subgroups of three mice were pooled, resulting in five biological replicates per experimental group. RNA was isolated from whole blood using the Gentra Purescript Blood RNA Isolation kit (Qiagen) and stored at −80°C in the presence of RNase inhibitors until processing. RNA quality was assessed using a bioanalyzer (Agilent Technologies, Santa Clara, CA) and by spectrophotometric analysis. cDNA was generated by amplification of the RNA using the Ovation RNA Amplification System V2 (NuGEN Technologies, Inc., San Carlos, CA). cDNA was fragmented to 50–100 bp, labelled with biotin, and hybridized to the Mouse 430_2 GeneChip high-density oligonucleotide array containing 39,000 mouse probe sets representing 34,000 genes (Affymetrix, Santa Clara, CA). Hybridization, staining and washing of all arrays was performed in an Affymetrix fluidics module. The dataset supporting the results of this article is available in the ArrayExpress database, accession number E-MTAB-3325 (http://www.ebi.ac.uk/arrayexpress).

Microarray data were analyzed using GeneSpring GX12.0 software (Agilent Technologies). Raw expression values were normalized by robust multi-array averaging, filtered to include only those probe sets with intensity values above the 20^th^ percentile, and baseline transformed to the median of the control samples. Statistical significance was determined by use of a one-way ANOVA with Welch correction for unequal variances. Criteria for differentially expressed transcripts included a *P*-value of <0.05 and at least a two-fold change relative to the control group. Differentially expressed genes were subjected to hierarchical clustering based on both transcripts and experimental condition.

### PCR array

Total RNA was isolated from skin biopsies collected from the site of inoculation in *B. burgdorferi* B515-infected and medium-inoculated control mice, as described above. For each experimental group, RNA from five animals was pooled, and a total of 1 μg pooled RNA was used as a template for first-strand cDNA synthesis. IFN-associated gene transcription was measured using the SABiosciences RT^2^ Profiler PCR Array PAMM-016: Mouse Interferon α,β Response kit (Qiagen), according to the manufacturer’s instructions.

### Real-time quantitative RT-PCR

Real-time quantitative RT-PCR was used to validate blood gene array data and to measure transcription of type I IFN-responsive genes in mouse skin biopsies. cDNA was synthesized from 1 μg of total RNA in 20-μl reaction mixtures containing 250 μM of each dNTP, 0.5 μg random primers, 5 U AMV Reverse Transcriptase (RT) enzyme, 10 U RNase inhibitor and 4 μL of 5X RT buffer (all from Promega). Reactions were performed at 42 °C for 75 min and terminated by heating at 94 °C for 5 min. cDNA was stored at −20°C until use.

PCR was performed using the following TaqMan Assays-on-Demand (Applied Biosystems, Grand Island, NY): *Camp* (cathelicidin antimicrobial peptide; Mm00438285_m1), *Ccr7* (chemokine (C-C motif) receptor 7; Mm01301785_m1), *Cxcl10* (chemokine (C-X-C motif) ligand 10; Mm00445235_m1), *Gbp1* (guanylate binding protein 1; Mm00657086_m1), *Ifit1* (interferon-induced protein with tetratricopeptide repeats 1; Mm00515153_m1), *Ifit3* (interferon-induced protein with tetratricopeptide repeats 3; Mm01704846_s1), *Ifnb1* (interferon-beta; Mm00439552_s1), *Irf3* (interferon regulatory factor 3; Mm00516779_m1), *Irf7* (interferon regulatory factor 7; Mm00516788_m1), *Mx1* (myxovirus (influenza virus) resistance 1; Mm00487796_m1), *Ngp* (neutrophil granule protein; Mm00476389_m1), *Stat2* (signal transducer and activator of transcription 2; Mm00490880_m1), and *Vcan* (*Cspg2*) (versican; chondroitin sulfate proteoglycan 2; Mm00490179_m1). All assays were performed in duplicate in 25 μL reaction mixtures containing TaqMan gene expression assay mix, FastStart universal probe master mix (Roche), and 2 μL cDNA (diluted 1:2) using the ABI 7900HT SDS sequence detection system (Applied Biosystems), according to the manufacturer’s instructions. As an internal control, the expression of murine β-actin was measured in duplicate for each sample using the TaqMan mouse endogenous control assay (*Actb*; Mm00607939_s1, Applied Biosystems). The ΔΔC_t_ relative quantitation method was used to calculate differential gene expression between groups [69].

### Statistical analysis

Statistical significance of differences between the mean RT-PCR fold-change values of experimental groups was determined using a one-way ANOVA with Tukey-Kramer’s post-hoc test (GraphPad Prism, version 5.03; GraphPad Software, San Diego, CA). A Fisher’s exact test was performed to determine significant differences in the numbers of infected tissues.
